# Ketone Bodies and SIRT1, Synergic Epigenetic Regulators for Metabolic Health: A Narrative Review

**DOI:** 10.3390/nu14153145

**Published:** 2022-07-30

**Authors:** Rossella Tozzi, Fiammetta Cipriani, Davide Masi, Sabrina Basciani, Mikiko Watanabe, Carla Lubrano, Lucio Gnessi, Stefania Mariani

**Affiliations:** 1 Department of Molecular Medicine, “Sapienza” University of Rome, 00161 Rome, Italy; 2 Department of Experimental Medicine, Section of Medical Physiopathology, Food Science and Endocrinology, “Sapienza” University of Rome, 00161 Rome, Italy; fiammetta.cipriani@uniroma1.it (F.C.); davide.masi@uniroma1.it (D.M.); sabrinabasciani@yahoo.it (S.B.); mikiko.watanabe@uniroma1.it (M.W.); carla.lubrano@uniroma1.it (C.L.); lucio.gnessi@uniroma1.it (L.G.)

**Keywords:** SIRT1, ketone bodies, ketogenic diet, β-OH-butyrate, obesity, epigenetic regulators, visceral fat, NAFLD

## Abstract

Ketone bodies (KBs) and Sirtuin-1 (SIRT1) have received increasing attention over the past two decades given their pivotal function in a variety of biological contexts, including transcriptional regulation, cell cycle progression, inflammation, metabolism, neurological and cardiovascular physiology, and cancer. As a consequence, the modulation of KBs and SIRT1 is considered a promising therapeutic option for many diseases. The direct regulation of gene expression can occur in vivo through histone modifications mediated by both SIRT1 and KBs during fasting or low-carbohydrate diets, and dietary metabolites may contribute to epigenetic regulation, leading to greater genomic plasticity. In this review, we provide an updated overview of the epigenetic interactions between KBs and SIRT1, with a particular glance at their central, synergistic roles for metabolic health.

## 1. Introduction

A huge number of factors are involved in controlling energy homeostasis, and nutrients are known to exert crucial epigenetic effects on metabolism.

A growing body of evidence supports the ketogenic diet (KD) as being effective in the management of diseases such as obesity [1]. Ketogenesis is a metabolic process leading to the production of ketone bodies (KBs)—acetoacetate (AcAc), beta-hydroxybutyrate (β-OHB), and acetone—which are a type of alternative fat-derived metabolic fuel for vital organs in states of nutrient deprivation. Ketogenesis occurs physiologically in the human liver throughout the day, producing KBs of up to 300 g per day. It is widely recognized that ketosis can generate neuroprotective [2,3] and beneficial metabolic effects [4,5,6]. The modifications evoked by ketosis, also obtained with exogenous ketogenic supplements [7], lead to an improved health status and delayed aging and related diseases through the epigenetic regulation of histones and RNA functions, and via the improvement of mitochondrial functions, antioxidant activities, and anti-inflammatory effects. Not surprisingly, the KD is providing data demonstrating its validity for the treatment of metabolic diseases.

Sirtuins (SIRTs) are class III nicotinamide-adenine-dinucleotide (NAD^+^)-dependent histone deacetylases (HDACs) deeply involved in the maintenance of genome stability, apoptosis, autophagy, senescence, proliferation, aging, and tumorigenesis. Among the seven known SIRTs, SIRT1 is the most studied member of the family. By deacetylating protein targets, SIRT1 has a key epigenetic role in the regulation of tissue homeostasis and is influenced by the convergence of several aspects, such as nutrition, metabolism, and chronobiology [8,9,10]. SIRT1 is known to modulate fat mobilization, muscle differentiation, food intake, and, in general, glucose and lipidic metabolism [11]. In addition to its effects strictly exerted in response to DNA damage, SIRT1 induces an increase in lifespan by protecting against the onset of age-related diseases such as diabetes mellitus [12], hepatic steatosis [13], cardiovascular diseases, atherosclerosis [14], osteoporosis [15], and neurodegenerative disorders [16]. The functioning of SIRT1 is linked to the availability of NAD+, and its expression is closely related to the presence of energy substrates being increased during fasting and decreased during overeating. After long periods of caloric restriction (CR), the increased expression of SIRT1 has been observed in white adipose tissue (WAT), which reduces fat storages and restores hormone levels, such as insulin and insulin-like growth factor 1 (IGF-1), altering the pace of aging [17].

Under CR and fasting, the production of KBs also increases and they can be used as energy fuel by the brain as an alternative to glucose. Furthermore, recent evidence has shed new light on the role of KBs in controlling protein acetylation, underlining the close link between KBs and SIRTs.

Thus, both nutritional ketosis and SIRT1 activation occur in response to caloric deprivation and mediate positive effects on cellular metabolism and senescence. The role of SIRT1 as an epigenetic regulator belonging to the category of histone deacetylases has been extensively studied, but recent lines of research have shown that KBs can also be considered as direct molecular drivers of epigenomic reprogramming and, thus, coordinate cellular functions through widespread covalent modifications of the lysine residues of regulatory histones. In this regard, given the close cooperative interaction between SIRT1 and KBs, it is likely that emerging tools of epigenetics can be used in the prevention, diagnosis, and treatment of diseases [18]. Herein, we aim to provide a review of the literature focusing on the epigenetic mechanisms of KBs and SIRT1 involved in metabolic outcomes, seeking to describe a direct link between these molecules during the ketogenic dietary management of obesity and its complications. Specifically, in our review, we first separately investigate the epigenetic regulation mediated by KBs and SIRT1, respectively, and then how these epigenetic modifications may act in the fine-tuning of adipose tissue remodeling, in the evolution of nonalcoholic fatty liver disease and in the resolution of low-grade systemic inflammation, a common aspect of all metabolic diseases.

## 2. The Epigenetics Regulation at a Glance

Epigenetics are defined as a set of modifications in gene expression without any changes in the gene sequence [19]. Although alterations in gene function can induce heritable phenotypic changes, this heritability across generations is less well established [20]. In any case, the alteration of gene expression patterns governed by epigenetics can result in diseases [21,22].

Hundreds of post-translational modifications (PTMs) have been found that can regulate cell differentiation, cell-specific gene expression, parental imprinting, X-chromosome inactivation, and genomic stability and structure. These include acetylation and deacetylation, methylation, and demethylation, phosphorylation, ubiquitination on the amino terminal or histone tail, and noncoding RNAs [19]. Several chromatin-modifying enzymes are responsible for adding and removing histone modifications contributing to the complex epigenetic process [19,23]. Moreover, the study of epigenetics is further complicated by the fact that the epigenome is tissue-specific, or even cell-specific, and that it changes over time [24]. Furthermore, numerous metabolic cofactors are required to support the catalytic activity of enzymes, and the availability and variation of the dietary-introduced metabolites influence epigenetic regulation [25,26].

**Figure 1:**
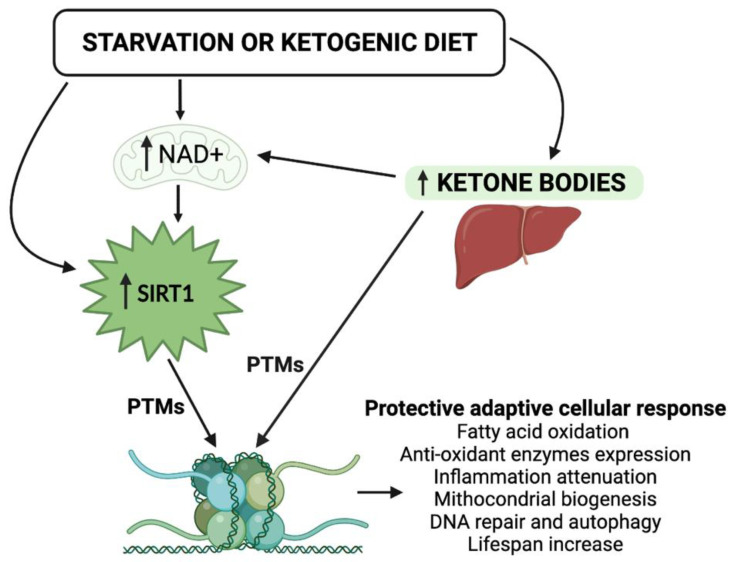
Schematic representation of the epigenetic activity of ketone bodies and SIRT1 in response to starvation or to the ketogenic diet. Synergistically, KBs and SIRT1 target both histone and nonhistone proteins and alter cellular metabolic programs. NAD, nicotinamide adenine dinucleotide; PTMs, post-translational modifications.

The intake of macronutrients and, more importantly, the amount, the timing, and the CR, while contributing to changes in cellular metabolism and the availability of NAD+ are crucial in inducing epigenomic adaptations. In patients with nutritional derangements resulting in metabolic disorders, such as obesity and diabetes mellitus, an association with DNA hypermethylation has been observed [27]. Interestingly, the KD positively modifies the redox state of the cell and modulates the activity of NAD+-dependent enzymes in deacetylation processes [28]. However, the majority of this evidence is derived from blood cells, limiting their interpretation in a clinical setting [24]. The study of the functional synergism between KBs and the NAD+-dependent deacetylase SIRT1—both involved in beneficial cellular metabolic processes—becomes of primary importance for understanding the epigenetic evolution of the KD, and of nutritional intervention in general (Figure 1). In view of the steady increase in metabolic disorders, including obesity, type 2 diabetes mellitus (T2DM), and cardiovascular diseases, we strongly believe that any nutritional therapeutic approach—with a particular focus on the KDs—should be adopted in order to reduce the burden of these diseases. Moreover, the in-depth study of epigenetic modifications induced by KBs and SIRT1 is of particular interest today, because it could represent an additional tool in the management of metabolic dysfunctions.

## 3. Ketone Bodies and Epigenetic Regulation

β-OHB, AcAc, and acetone are a set of fuel molecules that serve as an alternative energy source to glucose in the event of carbohydrate restriction. Other than being produced by the liver from fatty acids during periods of fasting or prolonged/intense physical activity, the endogenous production of KBs is promoted by consuming the KD, which consists of the intake of a very low amount of carbohydrates (generally, <50 g per day). In contrast to the pathological state of ketoacidosis, which can occur in decompensated diabetes, the nutritional induction of mild ketonemia has been proven to be beneficial in animal models, leading to improved metabolic profiles and neurological responses, as well as a prolonged lifespan [4,29]. Feeding elderly mice with a cyclic KD without reducing calory intake can decrease midlife mortality and prevent memory decline [30]. Furthermore, the administration of the KD to aged male mice preserves motor function and muscle mass and extends longevity [31].

In humans, the KD was initially used as a therapeutic option for its anticonvulsant effects in children with refractory epilepsy [32]; then, a very low carbohydrate ketogenic diet (VLCKD) was introduced for the treatment of obesity and its complications, such as insulin-resistance, type 2 diabetes [33], nonalcoholic fatty liver diseases (NAFLD) [34], and obstructive sleep apnea syndrome [35]. Moreover, apart from weight loss, the KD is widely used for the improvement of cardiovascular [28], rheumatological [36], neuronal [37], and, currently, cancer diseases [38,39].

Despite their beneficial use for several diseases, the systemic impact of the KDs is still only partially understood. Similar to what occurs during CR, the shift from carbohydrate to fat metabolism seen during the KD, and the subsequent oxidation of fatty acid, which increases the formation of KBs, appears to be responsible for the positive metabolic effects due to the intrinsic epigenetic function of KBs [40]. Indeed, β-OHB action is associated with protection against energy depletion, oxidative stress, inflammation, and apoptosis, and this could represent the downstream effect of KBs on antioxidant pathways in counteracting senescence [41].

The main histone PTMs induced by KBs are DNA methylation and histone phosphorylation, ubiquitination, and acetylation, which appear to be the key epigenetic mechanisms of β-OHB activity to modulate inflammation [42].

β-OHB-mediated hyperacetylation was observed under CR. This epigenetic activity modulates specific induction in gene expression, such as, for example, the Forkhead Box O3 A (FOXO3A), a family of proteins functioning as sensors of the insulin signaling pathway and regulators of longevity, which, in turn, regulates DNA transcription [30,43].

In addition to this direct mechanism, KBs ensure histone hyperacetylation through the inhibition of some deacetylase activities. Previous studies reported β-OHB as an endogenous inhibitor of class I histone deacetylase (HDACs) by competing for the catalytic site with butyrate, the structurally similar canonical HDAC inhibitor [9,43,44]. Shimazu et al. showed that β-OHB induces HDAC1, HDAC3, and HDAC4 (classes I and IIa) inhibition in renal cells driving the upregulation of FOXO3A transcription factor network genes, including the antioxidant catalase, the mitochondrial superoxide dismutase (SOD), and the metallothionein 2 [44].

In contrast, some findings did not confirm the prominent function of β-OHB as a histone deacetylase inhibitor [45], as well as the multilevel lysine hyperacetylation being questioned [42,46]. Even if the discussion on the HDAC inhibitory potential of β-OHB is still open, its overall effect on chromatin and mitochondrial protein acetylation could be attributable to the severe increase in intracellular acetyl-CoA and the cell potential intended as the NAD+/NADH ratio.

It should now be pointed out that the deacetylases-inhibiting activity of KBs may seem to be in contrast with the deacetylation activity carried out by SIRT1. To clarify this aspect, it is necessary to focus on the fact that KBs specifically inhibit histone deacetylases belonging to class I and IIa, and that this is not in conflict with SIRTs, which belong to the class III family of deacetylases. However, since fasting and nutritional ketosis act differently on different classes of HDACs, how these potentially opposing activities coordinate remains an open question and deserves further study.

Furthermore, the increase in NAD+ levels following KD activity is a factor available for the SIRT1 NAD+dependent activation, thus, further promoting deacetylation [47]. Interestingly, the metabolic shift towards fat oxidation and ketogenesis during starvation or during the KD is associated with initial mitochondrial stress characterized by increased levels of reactive oxygen species (ROS) and increased ratios of NAD+/NADH and adenosine monophosphate/adenosine triphosphate (AMP/ATP), as well as adenosine monophosphate/adenosine diphosphate (AMP/ADP). However, in the long-term, these “danger signals” provoke a protective and adaptive (hormetic) cellular response via the activation of SIRT1 and AMP-activated protein kinase (AMPK), and the consequences of the initial moderate metabolic stress include the upregulation of antioxidative and anti-inflammatory activities and an improved mitochondrial function [47].

As mentioned above, DNA methylation also represents a well know KB epigenetic action. Several studies have highlighted the antiepileptic efficacy of KBs, which, through altering DNA methylation, changes the expression of epileptogenic genes [48,49,50]. Some authors indicate KDs as exerting their disease-modifying effects through an adenosine-dependent epigenetic mechanism [51]. In general, similarly to hyperacetylation, the histone methylation status seems to be related to the pool of acetyl-CoA, which, together with glycine, is required for the synthesis of S-adenosylmethionine (SAM). Interestingly, a classical KD deprived of threonine (and, therefore, deficient in SAM, glycine, and acetyl-CoA) may paradoxically exacerbate seizures, as reported in epileptic rodent models [52]. In line with these findings, other studies showed that through DNA hypomethylation, KBs impact cardiac function. Indeed, hypomethylation affects the inflammatory functions of leukocytes related to cardiovascular risk through the modulation of adhesion/migration and soluble molecules [28], and global DNA hypomethylation has been observed in atherosclerotic lesions of mice, rabbits, and humans, and in hypertensive patients [28]. The hypomethylating activity of KBs may also have potential implications for the amelioration of metabolic diseases, in which DNA hypermethylation is frequently observed, as previously reported [27].

Recent findings suggest that β-OHB coordinates cellular functions via a novel epigenetic modification termed the β-hydroxybutyrylation, that integrates classic DNA methylation and PTMs, and includes specific histone lysines (up to 44 domains susceptible to this activity) and cellular proteins such as p53 [52]. As previously reported in renal cells and rodent models, exposure to high levels of β-OHB induces the β-hydroxybutyrylation of lysines 9 and 14 (H3K9/K14) in histone 3, whose increase leads directly to the upregulation of genes involved in hunger-sensitive metabolic networks that represent a fundamental mechanism of energetic and metabolic adaptation [53].

Finally, KBs also affect microRNAs (miRNAs). In volunteers treated with the KD, miRNAs directly targeted genes linked to nutrient metabolism, such as the mechanistic target of rapamycin (mTOR), peroxisome proliferator-activated receptors (PPARs), insulin, and cytokines, suggesting that the KD may modify miRNA expression, leading to the reduction in inflammatory interleukins such as IL-1β and IL-6, thus, mitigating neuroinflammation [3].

Ketone metabolism can affect the activity of SIRTs through the regulation of NAD+ availability, as well as that of other substrates. The epigenetic role of nutritional ketosis and, more generally, the relationship between macronutrients and gene expression need to be further explored.

## 4. SIRT1 and Epigenetic Regulation

SIRT1-related epigenetic mechanisms have been specifically investigated thanks to robust studies previously performed on yeast, which identified the *Sir2* gene as being responsible for lifespan extension [54]. The role of *Sir2* in the aging process has also been confirmed in higher organisms, such as Caenorhabditis elegans and Drosophila melanogaster [55,56], and several findings have led to the recognition of the *Sir2*-aging mechanism as an evolutionarily conserved system. Mammalian SIRT1 has been shown to exhibit similar activity to that of yeast *Sir2*, except for substrate deacetylase, which not only includes histones, but also key transcription factors such as p53, with an effect on tumorigenesis and maintaining normal cell growth, as well as FOXO [57,58].

Mainly located in the cell nucleus, in both prokaryotes and eukaryotes, SIRT1 can preferentially deacetylate lysine histone residues (H3K9 and H4K16) [59,60], as confirmed in SIRT1-deficient mouse embryonic fibroblasts characterized by hyperacetylated histones, heterochromatin formation, and transcription repression [61,62]. Expressed throughout all mammalian somatic and germ tissues, SIRT1 is responsible for aging-dependent global transcriptional changes caused by chromatin modification [11]. During starvation or a CR, SIRT1 regulates the expression of molecules involved in the response to fasting such as PPAR-γ, PPARγ-coactivator1-α (PGC1-α), FOXO, uncoupling proteins (UCPs), etc. Specifically, by regulating the expression of PPAR-γ, which exerts a pleiotropic role on inflammation, metabolism, endothelial function, oxidative stress, and apoptosis, SIRT1 ensures the balance of adipose stores in WAT. Fat storage is in part regulated by the activity of PPAR-γ on the aP2 gene, which encodes a protein that leads to fat storage. Interestingly, when mice are starved, SIRT1 is induced in WAT to suppress PPAR-γ by docking to the negative cofactors of the nuclear receptor and, at the same time, it binds the aP2 promoter, repressing its gene expression, ultimately leading to fat mobilizing in the blood stream [63]. These epigenetic changes lead to an increase in life expectancy, similar to what has been observed in mouse models deprived of adipogenesis (e.g., with insulin receptor deficiency) or with accelerated thermogenesis [64].

Metabolic diseases, such as obesity or liver steatosis, are accompanied by a high incidence of cancer diseases [65,66,67,68,69,70]. Through epigenetic activities, SIRT1 is known to play a significant role in the phenomena of carcinogenesis. However, the interplay between SIRT1 and DNA repair in cells is not completely understood yet, and, in some cases, results are even controversial [71,72,73,74,75,76]. For example, different SIRT1-induced modifications for DNA integrity preservation have been described both in normal and in cancer cells [8].

Most of the antitumor activity of SIRT1, however, is expressed through the containment of the metabolic syndrome. In fact, the increased life expectancy due to the epigenetic regulation of SIRT1 is due to the improvement of several metabolic and aging-related diseases, such as diabetes, NAFLD, cardiomyopathy, and neurodegenerative disorders [16,77]. More specifically, the antagonistic relationship between SIRT1 and NF-κB seems to be protective against hepatic steatosis and the metabolic energy balance under hypothalamic control [78]. Progression in fatty liver disease could be stopped by the AMPKa2-SIRT1-PPAR-α signaling pathway and by the SIRT1-mediated NLRP3 inflammasome suppression [79,80,81,82]. SIRT1 also exerts a beneficial effect against the development of atherosclerosis [14] and other diabetic complications. In this regard, it has been recently proven that tubular SIRT1 attenuates diabetic albuminuria by epigenetically suppressing claudin-1 overexpression in podocytes [83], whereas SIRT1 activators induce increased stress resistance in diabetic cardiomyopathy through the upregulation of the kinase ERK1/2 pathway and the sarcoplasmic reticulum Ca^2+^-ATPase SERCA2a [84].

Altogether, these findings suggest that many metabolic improvements are associated with the increased expression of SIRT1. Major metabolic changes, such as glycogen and fat mobilization, and the increase in gluconeogenesis and thermogenesis, along with changes in hormone levels (reduction in insulin and increase in glucagon, adipokines, and glucocorticoids levels), occur when nutritional conditions allow for an increase in the protective effects of SIRT1. In conclusion, the availability of the cofactor NAD+, closely related to nutritional intake and varying considerably between fasting and overeating states, activates SIRT1, which also mediates the action of other epigenetic modifiers.

## 5. Ketone Bodies and SIRT1 Share Common Metabolic Outcomes

Multiple pieces of evidence suggest that the protective effects of a CR are mediated by some gene expression profiles that reduce glucose metabolism and increase the use of alternative substrates [85]. The main function of KBs is to provide an alternative source of energy, particularly for the brain, to compensate for reduced blood glucose during food deprivation or fasting. Ketogenesis occurs mainly in the liver and starts from elevated free fatty acids (derived from lipogenesis as a consequence of reduced insulin concentrations) that metabolize into β-OHB and other KBs.

In patients with obesity, the administration of a VLCKD reduces blood pressure, reverses diabetic nephropathy [83], improves the lipid profile by switching from low-density lipoprotein (LDL) to high-density lipoprotein (HDL), ameliorates hepatic steatosis [5,86,87], etc. All these metabolic improvements are likely due to both weight loss and KBs effects, and this constitutes a bias in the attribution of the outcomes. However, as clearly demonstrated by the neuroprotective effects obtained in refractory epilepsy [7], the shift in the metabolic pathway and the blockage of glucose metabolism is responsible for the beneficial health effects in this pathology. Therefore, the study of ketosis in metabolic diseases is of considerable interest.

KBs and SIRT1 contribute to the same metabolic goals. Reflecting what happens during the establishment of ketosis, the adaptation to use different metabolic pathways in case of food deprivation or a lack of substrates is also guaranteed by SIRT1. SIRT1 exerts its activity primarily in response to a reduced caloric intake, and in patients with obesity, its plasma concentrations increase with fat loss [88]. Thanks to the deacetylation of PGC1-α, SIRT1 controls gluconeogenesis, the biogenesis of mitochondria, oxidative phosphorylation, and, finally, in coordination with PPAR-α, fatty acid oxidation [89]. The metabolic effects resulting from SIRT1 activation are expressed ubiquitously, but mostly in metabolic tissues such as the liver, adipose tissue, muscle, and the pancreas. SIRT1 has shown a negative correlation with both the percentage of adipose tissue and leptin, suggesting an active role in the regulation of fat stores [90]. In the liver, SIRT1 modulates the gluconeogenesis by deacetylating PGC1-α, FOXO,1 and the cAMP response element-binding (CREB)-regulated transcription coactivator 2 (CRTC2, also known as TORC2). Specifically, the deacetylation of FOXO1 and CRTC2 promotes a switch from the activation of early gluconeogenic genes to the activation of the late phase of gluconeogenesis genes when fasting is prolonged [91]. Furthermore, the repression of PPAR-γ in adipose tissue, the synthesis of UCP2 in pancreas β-cells, and the deacetylation of the liver X receptor (LXR) in the liver leads to fat mobilization, improved glucose-stimulated insulin secretion and an increase in the liver cholesterol efflux, respectively [92]. Consistent with these observations, KO mice for liver SIRT1 have shown hepatic steatosis and inflammation [93], and systemic SIRT1-null mice showed no adaptive feeding response to CR. Accordingly, transgenic mice overexpressing SIRT1 are protected against various metabolic disorders, including glucose intolerance, high-fat diet (HFD)-induced fatty liver, type 2 diabetes [94], age-induced cancer, osteoporosis, and cardiac hypertrophy [11]. Regarding bone health, the inverse relationship between SIRT1 and the bone formation inhibitor sclerostin present in bone tissue [15] and plasma [95] suggests a significant role for SIRT in regulating bone turnover, indicating SIRT1 as a target for the treatment of osteoporosis. In the heart, beneficial effects of SIRT1 against oxidative stress and aging have been observed [96]. Moreover, the use of β-OHB by a diabetic heart or in the case of heart failure represents an extraordinary example of an adaptive metabolic switch, which becomes an alternative response to stress during heart disease [97]. In line with these findings, circulating SIRT1 has shown an inverse association with epicardial fat thickness, the visceral fat depot of the heart [98].

**Figure 2:**
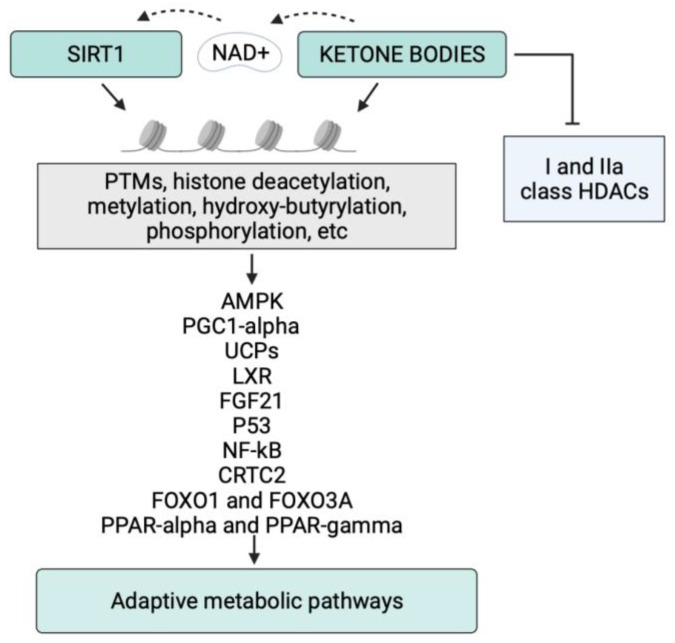
Schematic representation of the epigenetic control of KBs and SIRT1 on their targets. Post-translational modifications (PTMs) are key mechanisms for epigenetic regulation, which alters the activity of metabolic modulators. Abbreviations: NAD, nicotinamide adenine dinucleotide; AMPK, AMP-activated protein kinase; PPAR-alpha, peroxisome proliferator-activated receptor-α; PPAR-gamma, peroxisome proliferator-activated receptor-γ; PGC1-α, PPARγ-coactivator1-α; FOXO, Forkhead Box O; CRTC2, cAMP response element-binding (CREB)-regulated transcription coactivator 2; UCPs, uncoupling proteins; LXR, liver X receptor; FGF-21, fibroblast growth factor-21.

The relationship between SIRT1 and KBs and their shared metabolic outcomes justifies the interest in evaluating the epigenetic connections between KB formation and SIRT1 activation. In Figure 2, we report a schematic representation of the epigenetic control exerted by SIRT1 and KBs on their metabolic targets.

Some results suggest SIRT1 as a downstream effector of KBs. The incubation of HT22 hippocampal murine neurons with β-OHB has shown to result in a significant increase in SIRT1 and the upregulation of mitochondrial respiratory chain complexes [29]. Furthermore, a robust increase in SIRT activity was detected in murine hippocampal nuclear extracts after 3 weeks of KD treatment compared to the control, and the analysis of the nuclear expression showed an SIRT1 mRNA increase in the acute phase of ketosis [99]. These observations clearly indicated that treatment with the KD enhances SIRT1 activation.

On the other hand, as in a positive virtuous loop, SIRT1 supports the formation of KBs and the maintenance of ketosis. In fact, to facilitate the hepatic generation of KBs from fatty acids during long periods of energy deficit, SIRT1 cooperates with the CREB co-activator histone acetyltransferase p300/CBP, which interacts with PPAR-γ and PPAR-α in response to nutrient deprivation [91].

We can, therefore, summarize that, in the case of fasting, CR, or KD administration, the energy state of the cell depends on the simultaneous production of KBs, the increase in NAD+ and the enforced activity of SIRT1. Both KBs and SIRT1 then converge on PGC1-α, promoting the oxidation of fatty acids with a metabolic shift from glucose homeostasis. Moreover, it should be noted that, unlike glucose metabolism, in which four molecules of NAD are consumed, the generation of two moles of acetyl-CoA from one mole β-OHB consumes only one mole of NAD+ in the mitochondria, and the NAD+ saving allows to further stimulate the SIRT1’s obligate cofactor NAD+, rebalancing metabolic diseases [42]. KBs influence SIRT1 and substrates such as acetyl-CoA, HMG-CoA, and succinyl-CoA. HMG CoA is a precursor of cholesterol and an intermediate that is subsequently converted to β-OHB, the main KB present in the blood. This aspect may partly justify how epigenetic changes induced by KBs may be reflected in alterations in lipid metabolism and possibly improved dyslipidemia. Both CR and the KD, by increasing βOHB production, lead to similar metabolic results, contributing to an improved insulin resistance and systemic inflammation.

Globally, KBs and SIRT1 represent a dynamic and integrated metabolic node in human physiology (see Table 1). Although there are easy nutritional and pharmacological approaches to manipulate ketone metabolism, there is a need to further explore the impact of noncanonical signaling roles of KBs, including the regulation of PTMs that shape metabolic pathways to achieve therapeutic goals, especially in the view of the possible side effects of nutritional ketosis.

Below, we described the evidence of the reciprocal link between KBs and SIRT1 with particular focus on their epigenetic regulation in adipose tissue, the liver, and inflammation.

## 6. Epigenetic Activity of KBs and SIRT1 in Adipose Tissue Regulation

In humans, the reduction in SIRT1 mRNA transcription in visceral adipose tissue (VAT) is associated with obesity and hepatic steatosis [111]. To confirm these findings, studies on SIRT1 knock-out mice showed an increase in lipogenesis and a reduction in fat export from the liver [93]. A lower expression of SIRT1 may be responsible for the differentiation capacity of VAT-derived stem cells (ASC) in human obesity, fostering the expansion VAT [112], and visceral ASC isolated from subjects with obesity showed a reduction in the expression of SIRT1-6 when compared to both visceral ASC of normal weight subjects and ASC from subcutaneous adipose tissue [113]. These results emphasize the strong control that SIRT1 exerts on the physiopathology of adipose tissue. Interestingly, the genetic downregulation of SIRT1 in vivo causes the recruitment of macrophages to adipose tissue, while SIRT1 overexpression prevents adipose tissue macrophage accumulation due to a chronic HFD [114].

Some SIRT1 gene variations are associated with waist circumference (WC) and the waist–hip ratio in men with obesity [115]. Accordingly, Zillikens et al. recognized two common SIRT1 gene variants associated with a lower BMI and decreased risk of weight gain over time in a large Dutch population study [116]. Moreover, studies conducted on BMI-discordant monozygotic twins revealed downregulation in the NAD+/SIRT pathway expression in the twin affected by obesity [117].

As the primary storage site of lipid substrates, adipose tissue must be a primary contributor to the regulation of metabolism in food-deprived states. The early adaptive response to fasting enhances the mobilization and oxidation of endogenous fat, whereas glucose oxidation and energy expenditure are suppressed. SIRT1 is crucial for promoting the oxidation of fatty acids and supporting ketogenesis mainly through the activation of PGC1-α, which is a direct substrate of SIRT1 deacetylation. When fatty acids are elevated, acetyl Co-A is shunted in AcAc and does not enter the Krebs cycle. The same mechanism is induced by KD or fasting, or conditions characterized by glucose deprivation that usually arise following stressful events. The activation of the SIRT1/PGC1-α system represents a shift in the priorities of the cell away from pathways that stimulate growth towards pathways that reduce cellular stress and promote cell survival, including autophagy. The autophagic flux plays a critical role in hepatic and renal ketogenesis during starvation [102], and KBs in turn support autophagy both in vitro and in vivo [103]. Thus, autophagy may provide a direct link between the induction of a ketogenic fasting-like metabolic state and the activation of a cellular process that mutes cellular stress and neutralizes intracellular injurious pathways. Remarkably, KBs increase both PGC1-α and the expression of SIRT1, and Scheibye-Knudsen et al. demonstrated how a calorie-restricted HFD and, therefore, β-OHB, was able to enhance SIRT1 activity in mice and in a cellular model of Cockayne syndrome, decelerating premature aging [118].

Another critical aspect of fat metabolism concerns mitochondrial activity and thermogenesis regulated by brown adipose tissue (BAT). BAT is extremely important in fat storage and body weight maintenance. UCP1, which is located in BAT and active during thermogenesis to provide heat instead of ATP, plays a fundamental role in this context. The expression of UCP1 in BAT is controlled via SIRT1 [106]. Additionally, the consumption of the KD or a diet supplemented with ketone esters has been shown to induce UCP1 in the BAT of mice [4], and the administration of a 3-week high-fat KD to 8-week-old adult mice resulted in an increased expression in both PCG1-α and SIRT1 in BAT compared to mice fed regular chow. It should be highlighted that the isocaloric diets administered during these experiments did not induce a significant weight loss, suggesting that the overactivation of SIRT1 was attributable to ketosis rather than to the reduction in body weight [107]. Altered levels of SIRTs genes have been observed in the adipose tissue of individuals with obesity, indicating that a fat increase changes the expression of genes encoding SIRT1 [119]. On the other hand, a loss of fat increases SIRT1 circulating concentrations [88].

Given the profound connections between epigenetics and adipose metabolism, it is of particular interest to adopt new nutritional interventions that—via epigenetic modifications—may contribute to the improvement of pathological conditions depending on fat accumulation.

The following is a section on the relationship between epigenetics and ectopic fat in one of the most frequent obesity-related complications: nonalcoholic fatty liver disease (NAFLD).

## 7. Epigenetic Activity of KBs and SIRT1 against NAFLD

The number of incidences of NALFD is increasing due to the rise in the global prevalence of obesity, metabolic syndrome, and T2DM. NAFLD is characterized by excessive fat accumulation in the liver and can progress to nonalcoholic steatohepatitis with liver inflammation, hepatocyte damage, cell death, liver fibrosis, and, finally, cirrhosis.

Preliminary studies have demonstrated that hepatocyte-specific SIRT1 deletion impairs PPAR-α signaling and decreases fatty acid β-oxidation, resulting in a worse response to HFD, which has been associated with hepatic inflammation, endoplasmic reticulum stress, and NAFLD in mice [104]. We recently reported that plasma SIRT1 is inversely associated with NAFLD severity in a cohort of patients with obesity [120]. Currently, therapeutic mechanisms targeting NAFLD have been investigated, including the KD, which decrease liver fat and hepatocyte injury by suppressing inflammation and fibrosis [34]. In liver-specific SIRT1 knock-out mice, Li Yu et al. observed severe fatty liver disease and decreased levels of fibroblast growth factor 21 (FGF21) in the liver and circulation. KO animals showed a reduced hepatic expression of genes involved in fatty acid oxidation and ketogenesis, and an increased expression of genes controlling lipogenesis. By administering resveratrol or a synthetic SIRT1 activator (SRT1720), the transcriptional activity of the FGF21 promoter (−2070/+117) increased and, consistently, this increased the expression of genes that regulate fatty acid oxidation and energy expenditure, decreased fasting-induced steatosis, promoted browning of WAT, and reduced obesity [13]. Others have exalted the importance of the epigenetic intervention of KBs on the liver, showing that the administration of sodium butyrate plays a protective role on hepatic steatosis through the epigenetically regulated increase in FGF-21 induced through the inhibition of class I HDAC3 [109]. In support of this, the common belief that increasing the dietary fat intake invariably leads to fatty liver and prevents fat mass loss was recently proven wrong by an experiment showing that a normo-caloric ketogenic dietary regimen rich in fat inhibited de novo lipogenesis and induced fatty acid oxidation, leading to weight loss and a reduction in liver fat content [110]. More precisely, after fasting or during ketosis, the hepatic expression of SIRT1, together with that of PPAR-α, Jumonji-D3 histone demethylase (JMJD3), and FGF-21, increases. Upon FGF21 signaling, JMJD3 epigenetically upregulates global autophagy-network genes, leading to the inhibition of de novo lipogenesis and to fatty acid oxidation and NAFLD restraint. As already described above, autophagy is the mechanism adopted and shared by ketogenesis and SIRT1 at the liver level to preserve metabolic homeostasis [110]. Indeed, SIRT1 expression and autophagy induction are decreased in ob/ob mice and CR, as well as metformin, which alleviates hepatosteatosis by restoring SIRT1-mediated autophagy induction via an AMPK-independent pathway [121]. On the other hand, it seems that FGF21 supports the expression of SIRT1 by itself and the maintenance of the state of ketosis, as governing a positive autoregulatory loop involves PGC1-α during the adaptive starvation response [122,123]. Finally, increased FGF21 during the ketogenic state after fasting also supports LXR induction, showing a lipid and glucose metabolism improvement [108].

SIRT1 plays a role in the modulation of the liver cholesterol efflux also through the LXR deacetylation and activation in the nucleus [92], but several other compounds have been described to attenuate NAFLD and lipid metabolism through the AMPK/SIRT1/PGC1-α pathway [124]. The AMPK pathway, besides the inhibition of lipogenesis in adipocytes, the stimulation of glucose uptake in muscles, and the modulation of insulin secretion through pancreatic β-cells, promotes fatty acid oxidation and ketogenesis in the liver. This process requires PPAR-α activation and, as already reported, SIRT1 enhances β-oxidation and the maintenance of ketosis thorough cooperation with PPAR-α. Finally, once again, KBs in turn activate AMPK and support the disposal of inflammation while improving the intrahepatic fat discharge. This beneficial mechanism adds up to the promoter activity of SIRT1, which increases during the KD. Strategies to activate both KBs and SIRT1 could be used to treat fatty liver disease.

## 8. Epigenetic Activity of KBs and SIRT1 against Inflammation

The interaction between nutrition and immunity has been increasingly studied, showing that KBs and SIRTs may modulate immune cell function and inflammation. Acute inflammation normally resolves in an actively orchestrated series of molecular and cellular events that ensures the return to homeostasis after an inflammatory insult, a process regulated in part by endogenous specialized proresolving lipid mediators (SPMs). However, adipose tissue expansion in obesity promotes chronic inflammation, and its physiologic resolution is impaired. Obesity has been shown to be a state of SPM deficiency both in mice [125,126] and in the adipose tissue of human subjects [127].

The signaling role of β-OHB in inflammation is due to the fact that many cells of the immune system, including macrophages and monocytes, express hydroxycarboxylic acid receptor 2 (HCAR2) coupled to a G-protein (GPR), namely, GPR109A, of which β-OHB and butyric acid are the primary endogenous agonists [128]. Stubbs et al. demonstrated that short-term KD feeding changes the pool of innate cell immunity in VAT, resulting in decreased inflammation and improved metabolic health [129]. Using the RNA sequencing of adipose tissue immune cells, previous studies have shown that the KD expands the metabolically protective activity of γδ T cells with the consequent inhibition of inflammation, and that mice lacking γδ T cells showed impaired glucose homeostasis. These findings also suggest that γδ T cells are mediators of protective immuno-metabolic responses connecting fatty-acid-driven fuel use to the reduced adipose tissue inflammation [130]. The KD has even been proposed as an alternative care for systemic virus-induced inflammation in severe obesity [131]. However, the relationship between ketosis and inflammation sometimes appears to be misaligned. For example, although β-OHB exerts a primarily anti-inflammatory response [132,133,134], high concentrations of AcAc can trigger a proinflammatory response [135,136], and while prolonged nutrient deprivation has been shown to reduce inflammation [134], chronic ketosis in type 1 diabetes has been shown to yield a proinflammatory state [135,136].

Interestingly, some studies indicate that the anti-inflammatory effects of KBs are actually mediated by SIRT1 activity. In fact, β-OHB has been shown to activate AMPK through G-protein-coupled HCAR2, leading to a NAD+ generation increase and to the increased activity of SIRTs through the βHB/HCAR2/NAD+/SIRTs pathway [101]. Furthermore, the increased expression of SIRT1 leads to the induction of FOXO3 and NFkB, thereby evoking the expression of both the antioxidant genes and autophagy through the βHB/HCAR2/AMPK/SIRT1/FOXO3A/MnSOD and βHB/HCAR2/AMPK/SIRT1/NF-κB pathways, respectively [2,100]. At the neuroprotective level, autophagy appears to be triggered by mTORC-mediated SIRT1 inhibition during KD to reduce oxidation [100], and synergy between ketosis and SIRTs has been implicated against ischemic stroke by lowering ROS [137]. Evidence in vitro and in vivo shows that SPMs decrease crown-like structures in macrophages [105]. In line with these findings, the anti-inflammatory effects of SIRT1 have been observed at the adipose tissue level. Here, the genetic downregulation of SIRT1 in vivo results in the recruitment of macrophages, and the overexpression of SIRT1 prevents macrophage accumulation caused by a chronic HFD [114]. Accordingly, the time-dependent effects of the KD on resident immune cells in VAT have consequences for whole-body metabolic homeostasis [129]. Chronic unresolved inflammation plays a causal role in the development of advanced metabolic derangements, and KBs could be considered even a precious preventive measure for severe obesity complications [131,138].

## 9. Conclusions and Future Directions

Nutrition, energy metabolism, and gene expression are linked through the action of epigenetic modifiers. This review connects two emerging health-span metabolites, namely, β-OHB and the deacetylase SIRT1. Albeit the exact molecular mechanism of action of KBs still warrants further investigation, it is now well recognized that β-OHB may directly contribute to transcriptional regulation through epigenetic modulation, and, consequently, modulate inflammatory processes. A consequence of a reduced inflammasome is that KBs may have a role in the improvement of insulin resistance and related metabolic diseases. KBs are expected to interact with SIRT1, thereby activating antioxidant pathways. The study of SIRT1 and KBs in future research could provide insights into a better understanding of how to modulate the human inflammatory system and develop useful strategies to alleviate metabolic–inflammatory disorders. Another aspect that deserves consideration is whether there is an epigenetic code that can trigger the metabolic reprogramming of a CR or KD. In a virtuous reciprocal loop, both β-OHB and SIRT1 have a significant epigenetic potential to prevent and control several diseases, together improving health and life expectancy.

## Figures and Tables

**Table 1:** Principal epigenetic effects mediated by KBs and SIRT1 through their targets on metabolism.

Target	Main SIRT1 Effects	Main KBs Effects	Common Metabolic Outcomes
AMPK	Increase. Induction of FOXO3 and NF-kB evoking the expression of antioxidant genes and autophagy [2,100].	Activation through G-protein-coupled-HCAR2 leading to NAD+ increase [101]. Disposal of inflammation while improving intrahepatic fat discharge.	Anti-inflammatory effects; neuroprotection; protection against ischemic stroke.
PGC1-α	Deacetylation and activation. Support to the late phase of gluconeogenesis and fatty acid oxidation [91]. Autophagy [102].	Activation. Promotion of the oxidation of fatty acids with a metabolic shift from glucose homeostasis [103].	Adaptive starvation response. Increase in autophagic flux. Fat loss.
PPAR-α	Activation also through AMPK pathway. Improved β-oxidation of fatty acid, better response to HFD with decreased hepatic inflammation, endoplasmic reticulum stress, and NAFLD [104].	Increase. Reduction in inflammatory interleukins IL-1β and IL-6 [3,105].	Reduction in hepatic inflammation and NAFLD.
PPAR-γ	Suppression by docking to the negative cofactors of the nuclear receptor. Fat mobilization into the blood stream [63].	Repression. Reduction in inflammatory interleukins IL-1β and IL-6 mitigating neuroinflammation [3].	Fat loss.
UCP1	Increase. Induction in BAT after caloric restriction and nutrient deprivation [17,106].	Induction in BAT [4,107]. Expenditure of heat instead of ATP production.	Improved thermogenesis and energy expenditure.
LXR	Deacetylation and activation in the nucleus. Increase in the liver cholesterol efflux [92].	Activation after FGF21 induction. Glucose and lipid metabolism improvement [108].	Reduction in NAFLD.
FGF-21	Activation of transcriptional activity of the FGF21 promoter. Fatty acid oxidation and energy expenditure, decreased fasting-induced steatosis, promoted browning of WAT [13].	Increase in FGF-21 through the inhibition of class I HDAC3 [109]. Upregulation of global autophagy-network genes, inhibition of de novo lipogenesis.	Weight loss and reduction in liver fat content [110].
FOXO	Deacetylation and induction of FOXO1 in the liver with modulation of gluconeogenesis genes when fasting is prolonged [91]. Overexpression of FOXO3 with antioxidant effects [111].	Inhibition class I and II HDACs with the upregulation of FOXO3A transcription factor network genes [44] and modulation of DNA transcription [30,43].	Improved insulin signaling pathway and regulation of longevity.

Abbreviations: NAD, nicotinamide adenine dinucleotide; HDAC, histone deacetylase; BAT, brown adipose tissue; AMPK, AMP-activated protein kinase; PPAR-α, peroxisome proliferator-activated receptor-α; PPAR-γ, peroxisome proliferator-activated receptor-γ; PGC1-α, PPARγ-coactivator1-α; CRTC2, cAMP response element-binding (CREB)-regulated transcription coactivator 2; UCP, uncoupling protein; LXR, liver X receptor; HCAR2, hydroxycarboxylic acid receptor 2; FGF-21, fibroblast growth factor-21; FOXO, Forkhead Box O.
